# Supplementary material for: Sustained Delivery of a Shingles Subunit Vaccine Overcomes Age‐Related Declines in Humoral and Cellular Immunity Relative to Shingrix
Source: Adv Sci (Weinh). 2026 Jul 13;13(60):e76481. doi: 10.1002/advs.76481 (PMC13360122; doi:10.1002/advs.76481)
Supplement: Supplementary file 1 [file ADVS-13-e76481-s001.pdf]

Supplementary Information

Sustained Delivery of a Shingles Subunit Vaccine Overcomes Age-Related Declines in Humoral and Cellular Immunity Relative to Shingrix

Ye Eun Song, Jerry Yan, Ben S. Ou, Olivia M. Saouaf, Noah Eckman, Eric A. Appel

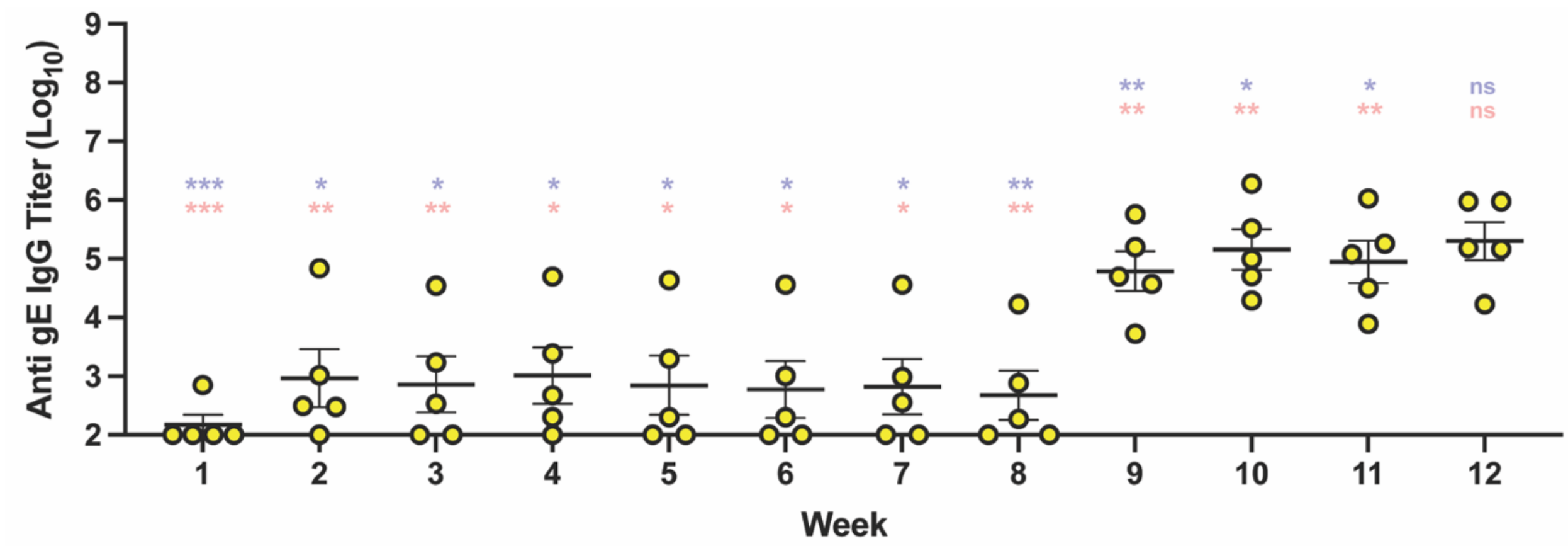

Fig S1. Total IgG response to subunit vaccine with alum and 3M-052 in 8-weeks-old mice. (*p < 0.05, **p < 0.01, ***p < 0.001)

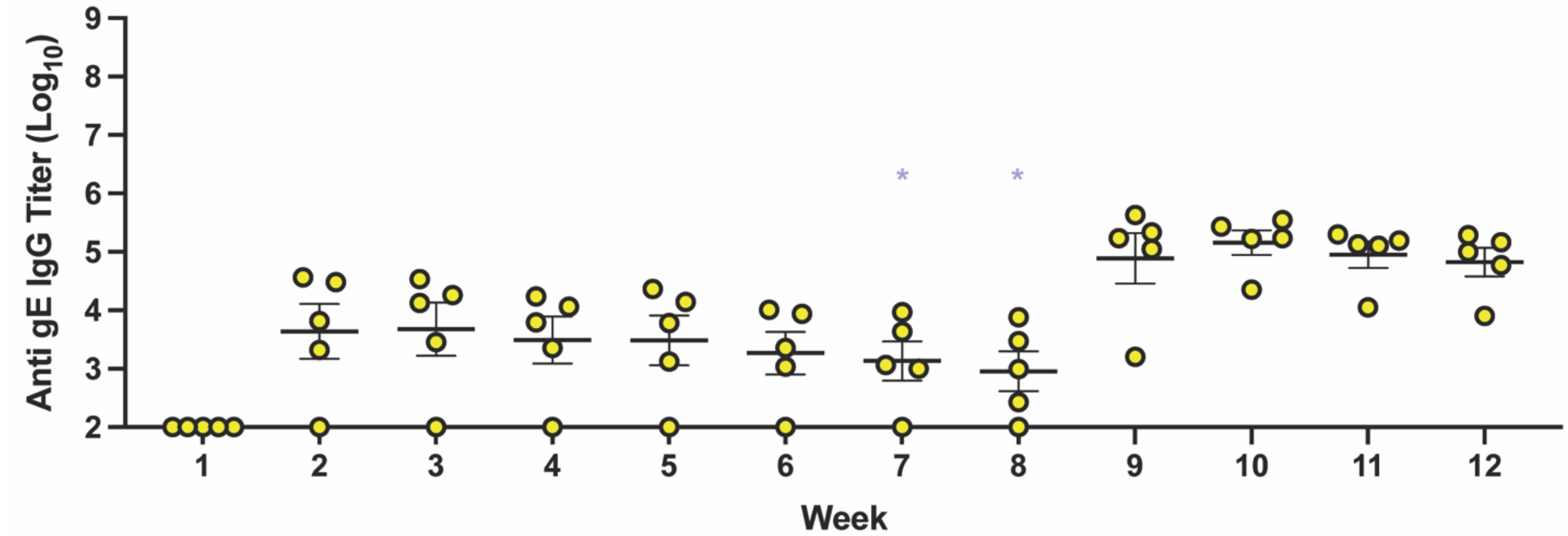

Fig S2. Total IgG response to subunit vaccine with alum and 3M-052 in 12-months-old mice. (*p < 0.05)

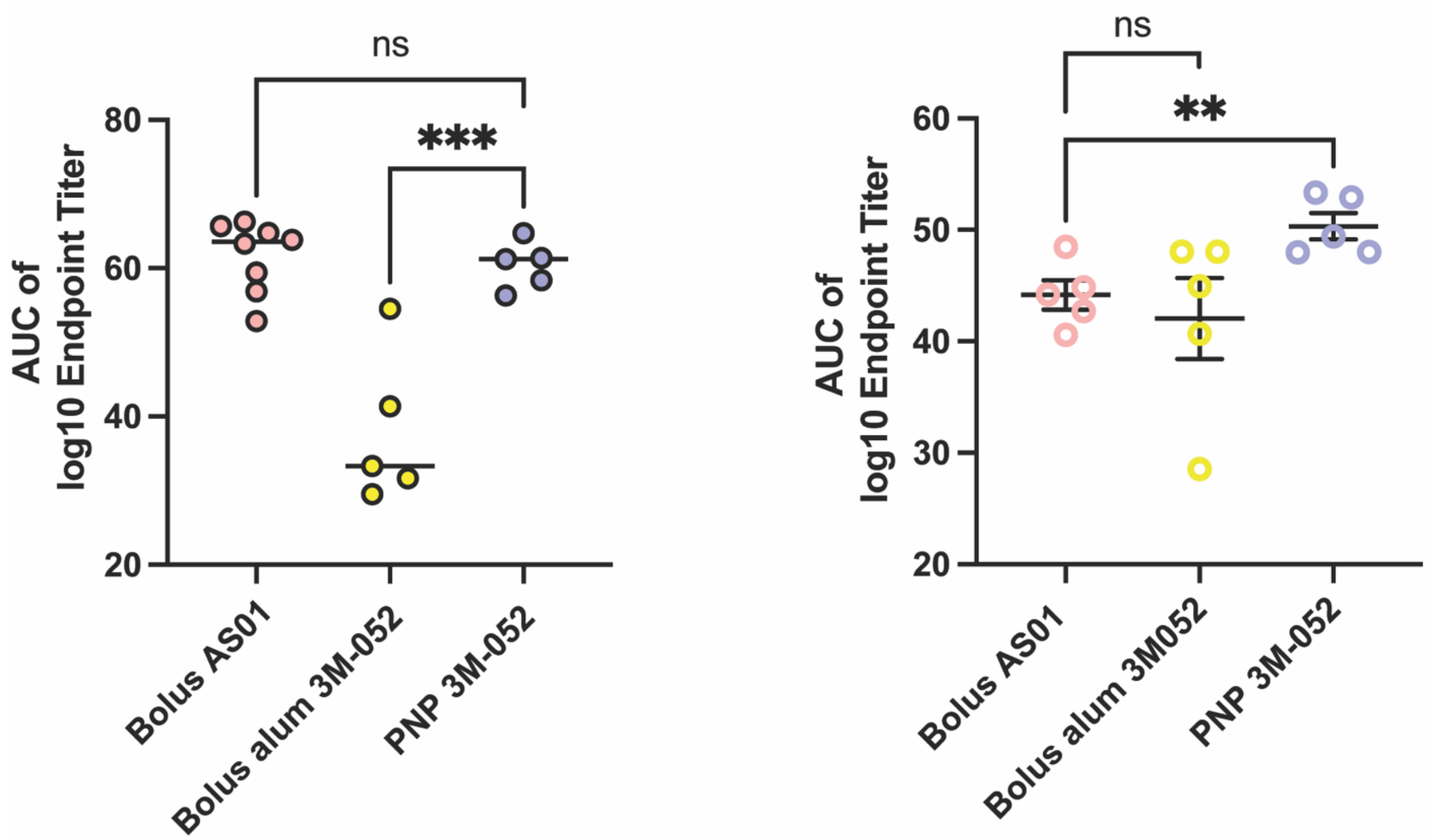

Fig S3. AUC from Fig S1 (left) and Fig S2 (right). (**p < 0.01, ***p < 0.001)

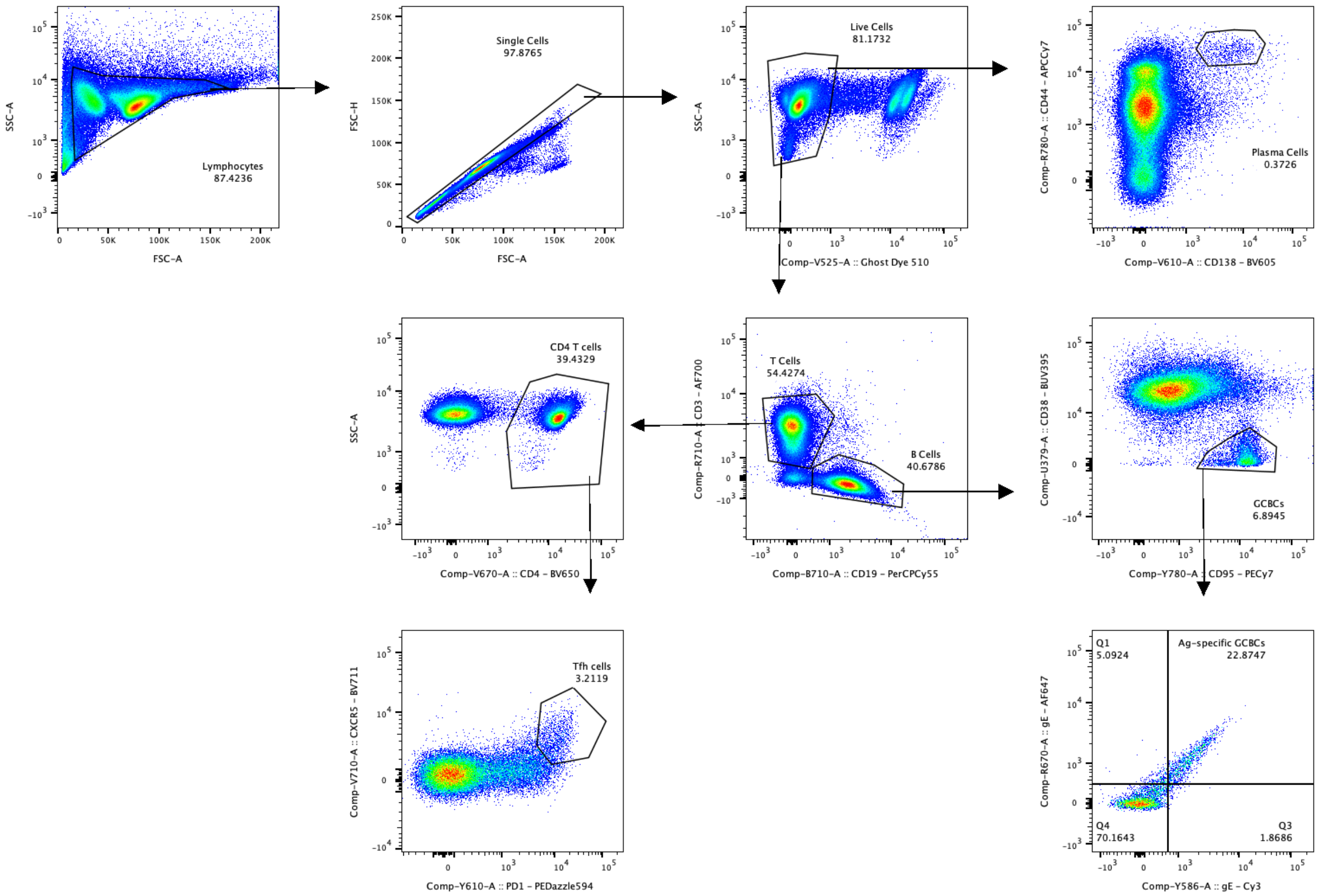

Fig S4. Sample gating scheme.
